# ID 221 - Iniciativas Relacionadas à ATS para Apoiar Magistrados na Tomada de Decisão Judicial em Saúde

**DOI:** 10.5327/2237-9622.2025.v34s1.221

**Published:** 2025-11-25

**Authors:** Isabela Porto de Toledo, Rafael Leite Pacheco, Camila Monteiro Cruz, Ana Luiza Cabrera Martimbianco, Carolina de Oliveira Cruz Latorraca, Cecília Menezes Farinasso, Patrícia do Carmo Silva Parreira, Roberta Borges Silva, Verônica Colpani, Rachel Riera

**Keywords:** judicialização da saúde, avaliação da tecnologia em saúde, ATS, NATJs, Rede NATJus, rede magistrados

## Abstract

**Introdução:**

Estima-se que, durante o ano de 2022, o sistema judiciário brasileiro tenha recebido por volta de 480.410 mil novas ações judiciais para fornecimento de medicamentos, dispositivos, procedimentos, ou outros processos relacionados à saúde no Sistema Único de Saúde (SUS) e saúde suplementar combinados. O Hospital Sírio-Libanês tem apoiado a tomada de decisão judicial em saúde no Brasil por meio do projeto Apoio Técnico-Científico à Tomada de Decisão Judicial em Saúde no Brasil (AD-Jus). O objetivo deste estudo é apresentar um conjunto de iniciativas que compõem este projeto, e que são relacionadas à Avaliação de Tecnologias em Saúde (ATS) e voltadas a apoiar magistrados e operadores do direito na tomada de decisão judicial em saúde.

**Método:**

Estudo descritivo analítico sobre as iniciativas de ATS conduzidas pelo Núcleo de Avaliação de Tecnologias em Saúde e Núcleo de Evidências do Hospital Sírio-Libanês (Nats/NEv-HSL) em parceria com o Departamento de Gestão das Demandas em Judicialização na Saúde do Ministério da Saúde e o Conselho Nacional de Justiça, por meio do Programa de Apoio ao Desenvolvimento Institucional do Sistema Único de Saúde (Proadi-SUS).

**Resultados:**

O conjunto inclui quatro iniciativas. (i) Blogue Rede NATJus (https://redenatjus.org.br/blog/) – blogue para disseminação de conteúdo científico, eventos e sínteses de evidências relevantes no contexto de judicialização em saúde. São três postagens semanais que se enquadram em uma das cinco categorias: "Judicialização", "Sistema e-natjus", "NATJus", "ATS" e "Agenda". A postagem mais relevante da semana é enviada, via e-mail, aos membros dos NATJus ou a qualquer cidadão cadastrado no blogue. Foram publicadas 449 postagens, sendo 223 no triênio 2018-2020, 115 no triênio 2021-2023 e 111 até 13 de setembro de 2024, no triênio 2024-2026. (ii) Consultas rápidas assíncronas, por meio de chat no canal Rede Magistrados (www.redemagistrados.hsl.org.br), permitindo que o Nats/NEv-HSL solucione dúvidas pontuais metodológicas ou clínicas demandadas por magistrados e relacionadas aos processos judiciais em saúde. Foram realizadas 37 consultas, sendo 7 no triênio 2021-2023 e 30 até 13 de setembro de 2024, para o triênio 2024-2026. (iii) Oficinas regionais presenciais para magistrados e assessores com foco em conceito de ATS e relevantes no contexto de judicialização da saúde. Até 13 setembro de 2024, foram realizadas 6 das 27 oficinas previstas para o triênio 2024-2026, com a participação de 95 operadores do direito. As oficinas foram sediadas nos Tribunais de Justiça ou nas Escolas Superiores de Magistratura das seguintes unidades federativas: Rondônia, Paraíba, Ceará, Tocantins, São Paulo e Santa Catarina. (iv) Glossário em ATS contemplando termos metodológicos e científicos utilizados em produtos científicos relevantes para o cenário de judicialização, com linguagem acessível e voltada para operadores do direito. O glossário está em elaboração, utilizando o método de, e seu protocolo está disponível em: https://osf.io/z384j/.

**Conclusão:**

O conjunto de iniciativas que vêm sendo conduzidas tem abrangência nacional e caráter estruturante, contribuindo para a sustentabilidade do SUS e sendo capaz de deixar um legado organizacional e processual mesmo após o término do período de execução de iniciativas. Como impacto esperado, é possível vislumbrar a disseminação da cultura da ATS entre operadores do direito como qualificadora do processo de decisão judicial, considerando as evidências científicas e priorizando tecnologias que claramente funcionam e são seguras em detrimento daquelas ineficazes, prejudiciais ou de eficácia/segurança incerta.

